# lncRNA MEG3 Inhibits the Proliferation and Growth of Glioma Cells by Downregulating Bcl-xL in the PI3K/Akt/NF-*κ*B Signal Pathway

**DOI:** 10.1155/2022/3729069

**Published:** 2022-07-11

**Authors:** Haibo Jia, Xiaoxiao Yan

**Affiliations:** ^1^Department of Neurosurgery, Handan Central Hospital, Handan, China; ^2^Department of Ophthalmology, Handan Central Hospital, Handan, China

## Abstract

This study was conducted to investigate the impact and mechanisms of lncRNA MEG3 on glioma cells. lncRNA MEG3 was lowly expressed in glioma cells as compared to noncancer cells. Overexpression of MEG3 significantly downregulated the expression of Bcl-xL, slightly upregulated the expression of NF-*κ*B p65 and I*κ*B*α*, and reduced the proliferation of glioma cells with increased apoptosis and the migration and invasion ability. Subsequently, glioma cells overexpressing MEG3 had less tumorgenicity in xenograft mouse models. It is likely that MEG3 induces apoptosis in glioma cells via downregulating the Bcl-xL gene in the PI3K/Akt/NF-*κ*B signal pathway to reduce the development of glioma.

## 1. Introduction

Gliomas form a heterogeneous group of tumors of the central nervous system (CNS) and are treated with a number of options such as steroid use, chemotherapy, radiotherapy, and surgery depending on their categories [1, 2]. However, the treatment response and overall survival rate are still very poor, particularly for patients with high-grade neoplasms [3]. Glioblastoma, the most aggressive malignant glioma, contains abundant microglia, which functions either as tumor suppressor (in the M1 phenotype) or tumor enhancer (in the M2 phenotype) depending on their polarization. Microglia plays an important role in the microenvironment that defines the cellular composition and molecular signatures of the glioma core [4, 5] and is considered to be important therapeutic targets for the development of new drugs or for the delivery of intraoperative near-infrared fluorescent dye for better surgical resection [6]. Furthermore, miRNA-mediated cross-talk between glioblastoma and microglial cells could reshape the microenvironment in glioblastoma to impact its progression. For example, miR-504, which is normally downregulated in glioblastoma, may be transferred via extracellular vesicles to microglial cells to polarize them to the M1 phenotype [7].

Gene therapies such as adenoviral vector- (Ad-) mediated gene therapy and oncolytic virotherapy have emerged as highly promising strategies for the treatment of malignant brain tumors due to recent progress in our understanding of the underlying cancer biology as well as improved techniques for genetic modification of potential therapeutics [8]. For example, Ad has been demonstrated to be able to achieve high levels of transgene expression with a safety profile for the treatment of retinal diseases and systemic brain metabolic diseases including glioma ([9]; J. [10]). The availability of these gene delivery technologies, such as Ad-based gene expression technology would pave ways to overexpress or knockdown targeted genes such as maternally expressed gene 3 (MEG3) to generate a therapeutic effect in glioma.

MEG3 is an imprinted gene located at the imprinted DLK1-MEG3 locus in human chromosome 14q32.3. The MEG3 gene encodes a long noncoding RNA (lncRNA) which is expressed in many normal tissues. However, the MEG3 gene expression is lost in many primary human tumors and tumor cell lines due to gene deletion, promoter hypermethylation, and hypermethylation of the intergenic differentially methylated region (J. [11]). MEG3 has been reported as a tumor suppressor gene since the reexpression of *MEG3* inhibits tumor cell proliferation ([12]; X. [13, 14]). Studies have shown that MEG3 can activate tumor suppressor p53 and dramatically stimulate the p53-dependent transcription from a p53-responsive promoter, inhibiting the proliferation of HCT116 cells [15]. MEG3 was also shown to inhibit proliferation and promote apoptosis in human glioma cell lines [16]. Therefore, MEG3 may have therapeutic potential for gliomas in gene therapy.

Several signaling pathways, such as the PI3K/AKT/NF-*κ*B signaling pathway, PTEN/PI3K/AKT signaling pathway, Wnt/beta-catenin pathway, and Sonic Hedgehog signaling pathway, are closely related to the occurrence, development, and drug resistance of cancers ([17]; H. [18–21]). Activation of PI3K/Akt/mTOR signaling pathways often occurs in various cancers [22] and is frequently associated with drug therapies [23], resulting in multidrug resistance [24]. These pathways may also be activated by a H1047R mutation in the catalytic subunit alpha (*PIK3CA*) of PI3K, leading to resistance to BRAF and MEK inhibitors in melanoma cells [25]. To better understand the impact of MEG3 on the proliferation, migration, and invasion of glioma cells, MEG3 was overexpressed in glioma cells and investigated for its effect on the biological characteristics of the cells and the expression of genes in the PI3K/Akt/NF-*κ*B signaling pathway. The findings would offer new clues to the use of MEG3 as a gene therapeutic agent for glioma.

## 2. Materials and Methods

### 2.1. Cell Culture

Human glioblastoma cell line U-251MG (cat no. 09063001, formerly U-373 MG) was obtained from the European Collection of Authenticated Cell Cultures and cultured in Minimum Essential Medium Eagle (EMEM, Gibco, Grand Island, NY, USA) + 2 mM glutamine + 1% nonessential amino acids + 1 mM sodium pyruvate (NaP) + 10% fetal bovine serum (FBS; Gibco) at 37°C in a 5% CO_2_ humidified atmosphere. Human astrocytes (HAs) were obtained from iXCells (San Diego, CA, USA) and cultured in an astrocyte medium (cat no. 10HU-035, iXCells) at 37°C in a 5% CO_2_ humidified atmosphere. Human tracheal epithelial cells (TECs) were obtained from ATTC (PCS-300-013, USA) and cultured in an airway epithelial cell basal medium (cat no. PCS-300-030, Sigma) at 37°C in a 5% CO_2_ humidified atmosphere.

### 2.2. Cell Transfection

To overexpress MEG3, U-251MG cells were seeded in the wells of 12-well plates at 1 × 10^5^ cells/well and grown to a confluency of 70%-80%. The cells were harvested, washed two times with PBS, and transfected with overexpression vector pMEG3 constructed using pLenti-CMV-MCS-EF1-GFP-T2A-puro as the backbone vector (https://www.alstembio.com/web/documents/Product_Specification_Sheet_LV111.pdf, Alstem, USA). In the vector, the expression of MEG3 is driven by the CMV promoter and EGFP is driven by the EF1 promoter, allowing the transfection to be visualized by green fluorescence. Mock transfection with the backbone vector was used as a control. Cells were transfected at a multiplicity of infection (MOI) of 100 using Lipofectamine 3000 (Thermo Fisher, USA) according to the supplier's instructions. After transfection, cells were incubated for 24 h in nonselective medium and transferred to a puromycin- (1.5 *μ*g/ml) containing medium for selection of transformed cells and clones. The transfection efficiency was analyzed using fluorescence microscopy.

### 2.3. RT-PCR

Total RNA was extracted from U-251MG cells using the TRIzol reagent (Themo Fisher Scientific, USA) according to the manufacturer's protocols. cDNA was transcribed from 1 *μ*g of total RNA using the HiFiScript first-strand cDNA Synthesis Kit according to the manufacturer's recommendations (Takara, Beijing, China). Quantitative real-time PCR reactions were carried out with cDNA (1 *μ*l), and the cycling conditions were 10 min at 95°C followed by 14 cycles, each one consisting of 15 s at 95°C and 4 min at 60°C, on a CFX Connect PCR system using TaqMan Pre-Amp Master Mix (Applied Biosystems). *β*-Actin was used as an internal reference to calculate the relative mRNA expression level using the 2-∆∆Ct method [26]. The primer sequences for PCR are presented in Table 1.

### 2.4. Western Blotting

Proteins were extracted from the cell lysate with a radioimmunoprecipitation assay (RIPA, cat. no. R0278, Sigma, USA) buffer containing protease inhibitors, quantitated using the BCA protein assay kit (cat. no. 23225, Thermo Fisher Scientific, USA) according to the manufacturer's instructions. After being denatured by boiling at 100°C for 5 min, 60 *μ*g of proteins was separated by 10% sodium dodecyl sulfate polyacrylamide gel electrophoresis (SDS-PAGE) and transferred to polyvinylidene fluoride (cat. no. VVLP04700, PVDF, Sigma, USA) membranes in an ice bath at constant voltage. The PVDF membranes were blocked with 5% nonfat milk in 1x Tris-buffered saline with 0.1% Tween 20 (TBST) buffer for 4 h at room temperature and incubated overnight with primary antibodies at 4°C. The antibodies used were against NF-*κ*B p65 (cat. no. AF1234, Beytime Biotech, Beijing, China,1 : 1500 dilution), Bcl-xL (cat. no. AB126, 1 : 2000 dilution), AKT1/2/3 (cat. no. AF0045, 1 : 1500 dilution), I*κ*B*α* (cat. no. AF2176, 1 : 1500 dilution), and *β*-actin (cat. no. ab8226, Abcam, USA). The membranes were washed six times with Tris-buffered saline (TBS) buffer, then added with horseradish peroxidase- (HRP-) conjugated goat antirabbit IgG (H+L) (cat. no. GtxRb-003-DHRPX, 1 : 1500 dilution, Affinity, USA). Immunoreactive bands were visualized with a chemiluminescence kit (cat. no. WP20005) from Themo Fisher Scientific, USA, in the dark. For quantification, bands were analyzed using Quantity One (v4.62) analysis software (General Electric, UK).

### 2.5. Cell Proliferation Assay

The cell proliferation was measured using the MTT (3-[4, 5-dimethylthiazol-2-yl]-2, 5 diphenyl tetrazolium bromide) assay as described [27]. Briefly, U-251MG cells were seeded into the wells of 96-well plates at a density of 5 × 10^4^ cells/well and cultured for 24, 48, and 72 h. 10 *μ*l of the MTT reagent (Sigma, USA) was added to each well, and cells were incubated for 2 h. The absorbance at 450 nm was then read using a microplate reader (Bio-Rad, USA) to measure the cell density according to the manufacturer's instructions. The experiments were independently repeated three times.

### 2.6. Transwell Cell Migration and Invasion Assays

Transwell cell migration and invasion assays were carried out to assess the migration and invasion ability of U-251MG cells based on the previously described protocols [28]. Briefly, cells were transfected with pMEG3 or controls, incubated for 48 h, pelleted by centrifugation at 500 × g for 10 min at room temperature, and resuspended in serum-free medium RPMI1640 medium. 2.0 × 10^4^ cells were inoculated into the upper chambers of Transwell inserts (8 *μ*m pore size; BD Bioscience, USA). The low chambers of the Transwell contained RPMI1640 medium with 10% FBS. The insert permeable membranes were either coated or not coated with Matrigel (Corning Life Sciences, USA) for assessment of cell invasion and migration, respectively. After 24 h incubation at 37°C, the cells remaining on the upper membranes were removed with a cotton wool, whereas the cells that had migrated or invaded through the membrane were stained with 2% crystal violet in 25% methanol/PBS, imaged, and counted in five randomly selected fields using an EVOS XL Core inverted microscope (Life Technologies, USA). The experiments were independently repeated three times.

### 2.7. Xenograft Assays

Eighteen BALB/c-nu athymic nude mice (Huafukang, Beijing, China) were randomly divided into three groups (*n* = 6). U-251MG cells (50 *μ*l, 5 × 10^6^) were planted subcutaneously on the dorsal side. The mice were allowed to grow for six weeks and were euthanized by inhaling carbon dioxide. The tumors were isolated and weighed. The animal study protocols were approved by the Animal Research Ethics Committee of Committee of Handan Central Hospital (approval no. 2019035).

### 2.8. Statistical Analysis

Statistical analyses were carried out with SPSS (version 19.0; SPSS, Inc., Chicago, IL, USA). Data are expressed as the means ± SD. Differences in means among groups were analyzed using one-way ANOVA followed by Tukey's post hoc test. *p* values ≤ 0.05 and ≤0.01 were considered statistically significant or highly significant, respectively.

## 3. Results

### 3.1. lncRNA MEG3 Expression Is Reduced in U-251MG Cells

**Figure 1:**
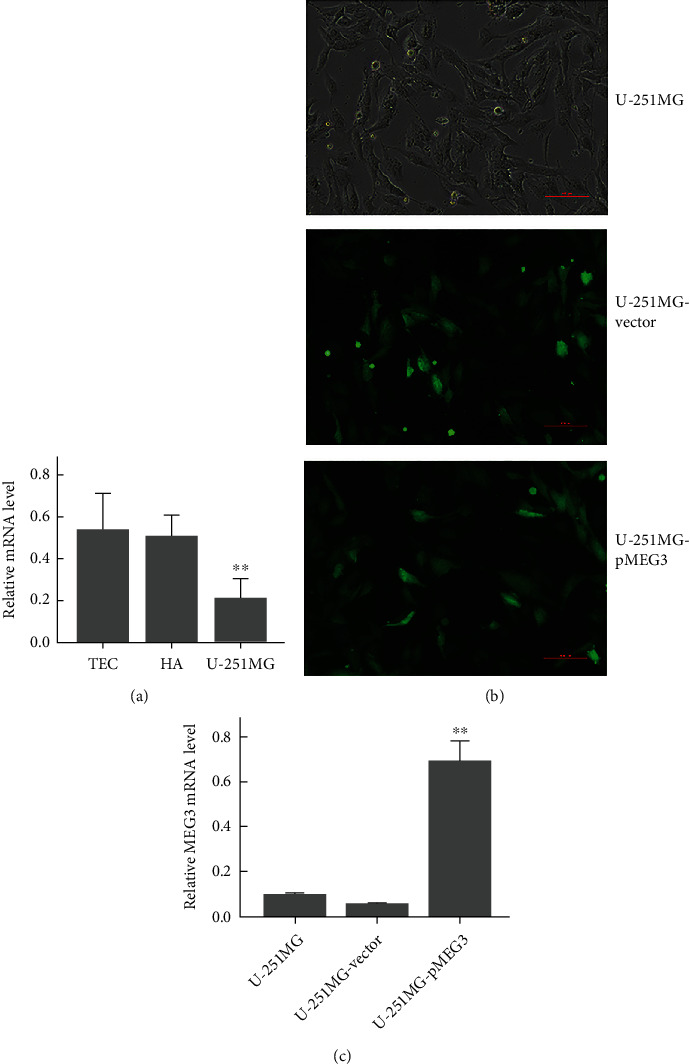
Expression of lncRNA MEG3 and transfection of U-251MG cells. (a) mRNA levels of lncRNA MEG3 in U-251MG cells, human astrocytes (HAs), and human tracheal epithelial cells (TECs); ∗∗ denotes *p* < 0.01 compared to Has and TECs. (b) Green fluorescence from U-251MG cells and U-251MG cells transfected with vector and pMEG3. (c) MEG3 mRNA levels in U-251MG cells after pMEG3 transfection. ∗∗ denotes *p* < 0.01 compared to U-251MG and U-251MG-vector.

The expression levels of MEG3 in U-251MG cells and noncancer cell lines HA and TEC were determined using RT-qPCR, and the results showed that MEG3 was significantly lower in U-251MG cells than in HAs and TECs (Figure 1(a)). To investigate the antitumor effect and mechanism of MEG3, a MEG3 overexpressing vector pMEG3 was constructed and transfected into U-251MG. After transfection with the vector and pMEG3, green fluorescence was observed in >70% cells under a fluorescence microscope, while no fluorescence was observed in nontransfected cells (Figure 1(b)), indicating that the transfection was successful and efficient. Furthermore, RT-qPCR assay showed that the MEG3 mRNA level was significantly upregulated after transfection as compared to nontransfected and vector-transfected cells (*p* < 0.01, Figure 1(c)).

### 3.2. lncRNA MEG3 Overexpression Suppresses Bcl-xL Expression in U-251MG Cells

**Figure 2:**
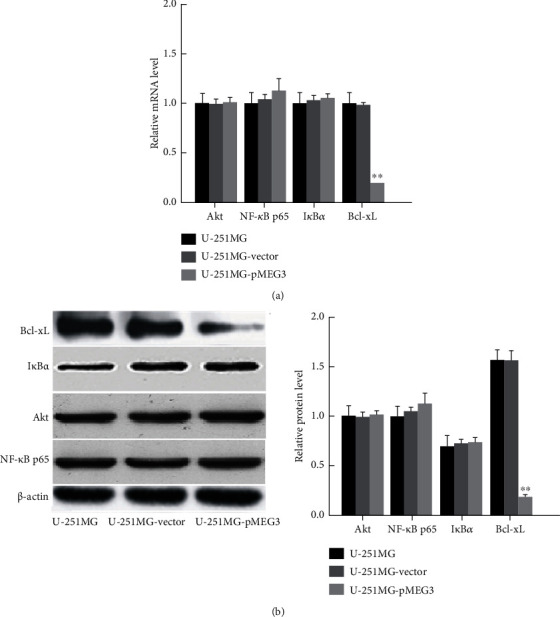
Expression of NF-*κ*B p65, I*κ*B*α*, Bcl-xL, and Akt following transfection of U-251MG cells with pMEG3 at mRNA and protein levels. (a) mRNA levels of NF-*κ*B p65, I*κ*B*α*, Bcl-xL, and Akt in U-251MG cells; (b) protein levels of NF-*κ*B p65, I*κ*B*α*, Bcl-xL, and Akt in U-251MG cells. Left panel: representative Western blots; right panel: protein contents. ∗∗ denotes *p* < 0.01 compared to U-251MG and U-251MG-vector.

Compared with the empty vector, transfection with pMEG3 slightly but insignificantly increased the expression of NF-*κ*B p65 and I*κ*B*α* at mRNA and protein levels, while the expression of Bcl-xL downstream the PI3K/Akt/NF-*κ*B signal pathway was downregulated significantly (*p* < 0.01, Figures 2(a) and 2(b)). There was no difference in the expression of Akt between the cells transfected with vector and pMEG3 (*p* > 0.05, Figures 2(a) and 2(b)).

### 3.3. lncRNA MEG3 Overexpression Reduces Cell Proliferation and Induces Apoptosis

**Figure 3:**
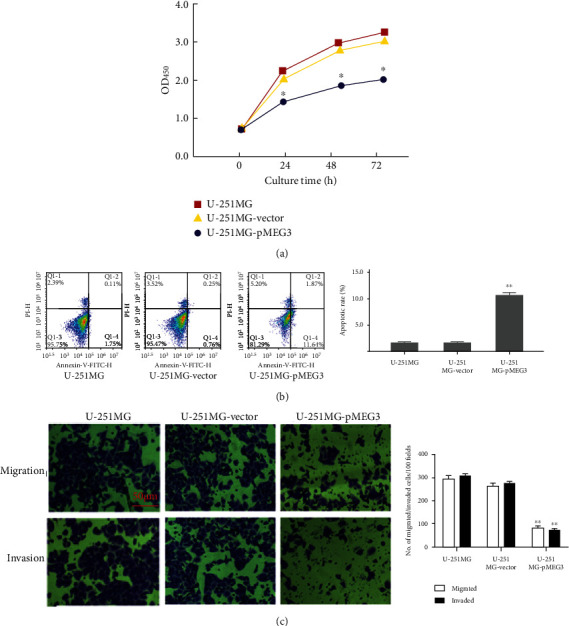
Proliferation, apoptosis, migration, and invasion of U-251MG cells after transfection with pMEG3. (a) CC8 assay results of U-251MG cells. (b) Apoptosis of U-251MG cells. Left panel: flowcytometry results; right panel: apoptotic rate. (c) Migration and invasion ability of U-251MG cells. Left panel: Transwell assay results; right panel: numbers of migrated and invaded cells. ∗ and ∗∗ denote *p* < 0.01 compared to U-251MG and U-251MG-vector.

After transfection with pMEG3, the cell proliferation was measured at different time points using a MTT kit. Compared with vector-transfected and nontransfected cells, the proliferation of pMEG3-transfected cells was significantly slower at 24, 48, and 72 h after transfection (*p* < 0.01, Figure 3(a)). Flow cytometry showed that there were significantly more apoptotic cells after transfection with pMEG3 as compared with controls (*p* < 0.01, Figure 3(b)).

### 3.4. lncRNA MEG3 Overexpression Inhibits Cell Migration and Invasion

The effect of MEG3 overexpression on cell migration ability was examined using Transwell assays. After transfection with pMEG3, the migration ability of U-251MG cells was significantly reduced as compared with the vector- or nontransfected cells (*p* < 0.01, Figure 3(c)). Similarly, the Transwell assay showed that pMEG3 reduced significantly the invasive ability of the pMEG3-transfected U-251MG cells with significantly lower number of cells invaded through the gels as compared with the controls (*p* < 0.01, Figure 3(c)).

### 3.5. lncRNA MEG3 Overexpression Reduces the Proliferation of U-251MG Cells in Xenograft Model

**Figure 4:**
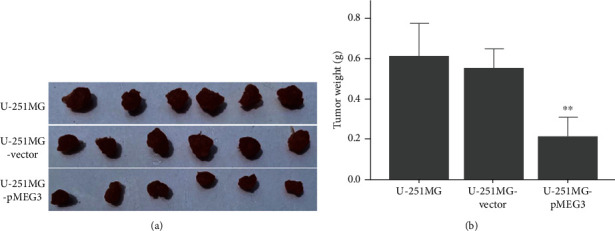
Growth of tumors in mice engrafted with vector and pMEG3-transfected U-251MG. (a) Photos of isolated tumors at five weeks after engraftment; (b) weight of tumor. ∗∗ denotes *p* < 0.01 compared to U-251MG and U-251MG-vector.

We then investigated the effect of MEG3 on the proliferation of U-251MG cells in the xenograft models of mice. After being engrafted with U-251MG, tumors were developed in the nude mice and grew faster in mice engrafted with U-251MG or U-251MG-vector than with U-251MG-pMEG3. About four weeks after engraftment, tumor necrosis was seen in the control group, resulting in bleeding of the tumor and the darkening of tumor color. Six weeks after engraftment, the tumors were isolated and weighted. The result showed that the tumors isolated from mice engrafted with pMEG3-transfected U-251MG cells were significantly smaller than those from mice engrafted with vector-transfected U-251MG or U-251MG cells (Figure 4).

## 4. Discussion

Gliomas are the most common primary intracranial tumor, representing over 80% of malignant brain tumors [29]. Currently, there are increasing interests in developing targeted gene therapy for this tumor. MEG3 has been shown to have antitumor activity in different cancer cells, such as breast, liver, glioma, colorectal, cervical, gastric, lung, ovarian, and osteosarcoma cancer cells [30]. Previously, it was shown that MEG3 represses tumor through regulating the major tumor suppressor genes p53 and Rb, inhibiting angiogenesis-related factor, or controlling miRNAs [15, 31, 32]. In this study, we show that MEG3 is downregulated in the glioblastoma cells and overexpression of MEG3 could inhibit the growth and reduce the migration and invasion ability of the glioblastoma cell line U-251MG. It also increased apoptosis of U-251MG and reduced its tumorigenicity in mice. Molecular analysis showed that overexpressing MEG3 results in reduced expression of Bcl-xL downstream the PI3K/AKT signal pathway.

Recently, the accumulating scientific evidence indicates that there is a clear association between lncRNAs and tumorigenesis [33], and lncRNAs may be classified based on their expression patterns and functions at the cellular level into tumor suppressor genes and oncogenes [34]. For example, using both in silico approaches and in vitro analyses, lncRNA LINC00483 is found to be a tumor (colorectal cancer) repressor functioning as a miRNA sponge [35]. The MEG3 gene is located in human chromosome 14q32.3 within the DLK1-MEG3 locus [36] and composed of 35 kb size and made up of ten exons [12], encoding an approximately 1.6 kb lncRNA (X. [37]). Previous studies showed that compared to adjacent normal tissues, MEG3 is downregulated in a number of cancers such as breast cancer (J.J. [38]), glioma cells [16], colorectal cancer [39], and cervical cancer (R. [40]). We compared the expression of MEG3 in the glioblastoma cell line U-251MG with noncancer cells HAs and TECs and found that the mRNA level of MEG3 in the glioblastoma cells is significantly downregulated. This is consistent with previous studies. Previously, the reduced MEG3 was attributed to the methylation at the MEG3 promoter CRE site, and menin could activate lncRNA MEG3 to allow binding of the transcription factor cAMP response element-binding protein [41]. However, the mechanism underlying the reduced MEG3 expression in the glioblastoma cells is unclear.

Noncoding RNAs, including lncRNAs, have been found to play a multitude of roles in cancer and function as molecular decoys, scaffolds, enhancers, or repressors, which control glioblastoma cells to differentiate, proliferate, and invade at the expression and/or epigenetic levels [42]. Recently, new data show that there are the cross-regulations between lncRNAs and small noncoding RNAs that affect the phenotypic diversity of glioblastoma subclasses [43]. In addition, circular RNAs (circRNAs) are also involved in the regulation of gene expression (acting as microRNA (miRNA) or RNA binding protein (RBP) sponges) ([44, 45]; M. [46]), and some circRNAs, such as circSMARCA5 and circHIPK3, are found to be good diagnostic biomarkers for glioblastoma multiforme [47], suggesting that circRNAs may also interact with MEG3 to play a role in glioblastoma progression.

Our data from Transwell and proliferation experiments showed that overexpression of MEG3 inhibits the proliferation, migration, and invasion of glioma cells. The PI3K/Akt/NF-*κ*B pathway is closely related to these biological properties (Y. [48, 49]). For example, AnnexinA5 is shown to activate the PI3K/Akt/NF-*κ*B signaling pathway via targeting Snail to promote cell migration and invasion in vitro and tumorigenicity of glioma cells in nude mice [48]; 14-3-3*β* promotes the migration and invasion of human hepatocellular carcinoma cells through the PI3K/Akt/NF-*κ*B signaling pathway [50]. The signaling pathway is also associated with apoptosis, proliferation, survival, and differentiation of cancer cells [51]. NF-*κ*B is an active transcription factor in cancer cells and induces the transcriptions of target genes to mediate EMT, invasion, angiogenesis, metastasis, and proliferation, leading to increased apoptosis ([52, 53]; Baldwin 1996). We profiled the expression of key proteins in the signaling pathway at mRNA and protein levels and found that NF-*κ*B p65 and I*κ*B*α* expressions are upregulated slightly and Bcl-xL expression is downregulated significantly after overexpressing MEG3 in U-251MG cells. Studies show that the activation of NF-*κ*B p65 could promote the production of proangiogenic factors, leading to increased blood supply of tumor cells and tumor growth [54–56]. Bcl-xL is one of the common antiapoptotic proteins that promote cell survival [57]. Downregulation of Bcl-xL could result in a downregulation of multiple metabolic genes, including genes that are involved in both glycolysis and oxidative phosphorylation [58]. Bcl-xL could also prevent apoptosis by sequestering proforms of death-driving cysteine proteases called caspases (a complex called the apoptosome) or by preventing the release of mitochondrial apoptogenic factors such as cytochrome c and AIF (apoptosis-inducing factor) into the cytoplasm [59]. Therefore, tumor cell proliferation would be reduced and apoptosis would be initiated when Bcl-xL is downregulated as observed in this study.

In animal experiments, the tumors derived from cells overexpressing MEG3 were significantly smaller as compared to controls, suggesting that MEG3 reduces the tumorigenicity of the cells. Since in vitro experiments demonstrated that overexpressing MEG3 reduces the cell proliferation and increases apoptosis, it is likely that the reduced tumorigenicity resulted from attenuated cell viability.

It should be noticed that in this study, only one cell line U-251MG was used for bioassay and expression analysis. Although U-251MG (formerly known as U-373 MG) was derived from malignant glioblastoma and is widely used as an experimental model of glioblastoma (H. [60, 61]), there might be difference among the cell lines with respect to gene expression and biological behavior ([62]; N. [63, 64]). Therefore, it is likely that results obtained from U-251MG cells might not be well representative for glioma and should be dealt with caution and validated with more in vitro and in vivo studies. There are other limitations in the study. Only one expressing vector was used for MEG3; therefore, it was not able to investigate the dose-inhibition response of the gene in glioma. In addition, the mechanism leading to RNA MEG3 downregulation in U-251MG was not investigated.

## 5. Conclusion

Our results demonstrate that lncRNA MEG3 inhibits the proliferation and growth of glioma cells in vitro and in vivo. This inhibition is likely achieved by downregulating the expression of Bcl-xL in the PI3K/Akt/NF-*κ*B signal pathway. Our findings suggest that lncRNA MEG3 may be further explored as a potential new gene therapeutic agent for the treatment of glioma. However, the data were obtained with a single cell line; more in vitro and in vivo studies are needed to validate the conclusions.

## Figures and Tables

**Table 1:** Sequences of the primers.

Primers	Forward	Reverse
MEG3	5′-AGACGGCGGAGAGCAGAG	5′-CACATTTATTGAGAGCACAGTGG
AKT	5′-TGGACTACCTGCACTCGGAGAA	5′-GTGCCGCAAAAGGTCTTCATGG
NF-*κ*Bp65	5′-GTCTTCGTGCTCGGTGATG	5′-AGGACCTCTGACCCAAATG
I*κ*B*α*	5′-AACCTGCAGCAGACTCCACT	5′-ACACCAGGTCAGGATTTTGC
Bcl-xL	5′-GTAAACTGGGGTCGCATTGT	5′-TGCTGCATTGTTCCCATAGA
*β*-Actin	5′-CATCCCCCAAAGTTCACAAT	5′-AGTGGGGTGGCTTTTAGGAT

## Data Availability

The datasets used during the current study are available from the corresponding author on reasonable request.

## Abbreviations

lncRNA: Long noncoding RNA
qRT-PCR: Quantitative reverse transcription PCR
PCR: Polymerase chain reaction
MTT: 3-(4,5-Dimethylthiazol-2-yl)-2,5-diphenyltetrazolium bromide
CNS: Central nervous system
Ad: Adenoviral vector
MEG3: Maternally expressed gene 3
NaP: Sodium pyruvate
HA: Human astrocyte
FBS: Fetal bovine serum
TEC: Tracheal epithelial cell
MOI: Multiplicity of infection
RIPA: Radioimmunoprecipitation assay
SDS-PAGE: Sodium dodecyl sulfate polyacrylamide gel electrophoresis
PVDF: Polyvinylidene fluoride
TBST: Tris-buffered saline with 0.1% Tween 20
TBS: Tris-buffered saline
HRP: Horseradish peroxidase
SD: Standard derivation
ANOVA: Analysis of variance.
